# Exploring the Potential of Ultrasound Therapy to Reduce Skin Scars: An In Vitro Study Using a Multi-Well Device Based on Printable Piezoelectric Transducers

**DOI:** 10.3390/bioengineering10050566

**Published:** 2023-05-09

**Authors:** Simone Riis Porsborg, Hubert Krzyslak, Malgorzata K. Pierchala, Vincent Trolé, Konstantin Astafiev, Rasmus Lou-Moeller, Cristian Pablo Pennisi

**Affiliations:** 1 Regenerative Medicine Group, Department of Health Science and Technology, Aalborg University, DK-9260 Gistrup, Denmark; sriis@hst.aau.dk (S.R.P.); hubert.krzyslak16@gmail.com (H.K.); 2 CTS Ferroperm Piezoceramics, DK-3490 Kvistgaard, Denmark; malgorzata.pierchala@gmail.com (M.K.P.); vincent.trole@ctscorp.com (V.T.); k_astafiev@hotmail.com (K.A.); rasmus.moeller@ctscorp.com (R.L.-M.)

**Keywords:** therapeutic ultrasound, skin scarring, flexible piezoelectric material, extracellular matrix

## Abstract

Excessive skin scarring affects over 100 million patients worldwide, with effects ranging from cosmetic to systemic problems, and an effective treatment is yet to be found. Ultrasound-based therapies have been used to treat a variety of skin disorders, but the exact mechanisms behind the observed effects are still unclear. The aim of this work was to demonstrate the potential of ultrasound for the treatment of abnormal scarring by developing a multi-well device based on printable piezoelectric material (PiezoPaint™). First, compatibility with cell cultures was evaluated using measurements of heat shock response and cell viability. Second, the multi-well device was used to treat human fibroblasts with ultrasound and quantify their proliferation, focal adhesions, and extracellular matrix (ECM) production. Ultrasound caused a significant reduction in fibroblast growth and ECM deposition without changes in cell viability or adhesion. The data suggest that these effects were mediated by nonthermal mechanisms. Interestingly, the overall results suggest that ultrasound treatment would a be beneficial therapy for scar reduction. In addition, it is expected that this device will be a useful tool for mapping the effects of ultrasound treatment on cultured cells.

## 1. Introduction

Skin scarring is a biological process that occurs as a result of tissue repair in mammals. It is estimated that approximately 100 million patients develop scars each year in high-income countries alone [1]. In many cases where traumatic injury or surgery damages the deep dermis, excessive scarring may happen as a result of an abnormal tissue response. In these cases, an exaggerated healing process occurs, causing the scar to thicken (hypertrophic scar) or develop into a lesion that grows beyond the margins of the original injury (keloid). Although hypertrophic scars and keloids differ in their histopathologic features and clinical appearance, they are both caused by an overproduction of collagen, which continues to be deposited at the site of the wound [2]. The main cells mediating extracellular matrix (ECM) protein turnover are dermal fibroblasts, which are thought to be dysregulated in abnormal scarring disorders, but the exact pathogenesis is still not fully understood [3]. While some scars may be considered as trivial aesthetic skin lesions, abnormal scars can cause a variety of problems ranging from itching, soreness, and pain at the site of injury to systemic effects, such as sleep disturbances, anxiety, depression, and impairment of daily activities.

The conservative approach for managing scars is the so-called leave-alone therapy, in which the scar is observed for a year before a decision is made on the further course of treatment, but this may have undesirable consequences for the patient [2]. Passive or active treatment options are currently available as alternatives. Passive treatments are usually non-invasive, such as the application of a compressive silicone patch over the scar [4]. They are affordable but require continuous patch application for at least three months to a year, and there is a high risk of scar recurrence [5]. Active therapies, on the other hand, are usually invasive and consist of either surgery or the use of laser radiation. These treatments can be expensive and carry the risk of side effects associated with a long recovery time [6].

Alternative methods are being sought to reduce the time and cost of treatment. In this direction, ultrasound-based therapy has shown to be viable option for the treatment of abnormal scarring. For example, the use of ultrasound has been shown to reduce the growth of keloids or postoperative scars and to facilitate the penetration of anti-scarring drugs into the dermis [7]. In general, within the field of dermatology, ultrasound-based therapies have been used to treat a variety of wound healing disorders, as described in recent literature reviews [8,9]. For example, studies have shown that ultrasound can enhance antibiotic penetration into bacterial biofilms [10] and promote the healing of venous leg ulcers [11,12] and chronic diabetic wounds [13]. The improvement in wound healing rate appears to be associated with the modulation of macrophages and fibroblasts’ activity, which are critical for successful wound closure [14].

One of the desirable features of an ultrasonic therapy device for dermatology is the ability to adapt the ultrasound transducers to the body surface in order to increase the efficiency of the treatment [15]. An attractive material for the fabrication of such ultrasonic transducers is PiezoPaint^TM^, a flexible piezoelectric material composed of commercially available lead zirconate titanate (PZT) ceramic and a polymer matrix [16,17]. It is compatible with commercial printing techniques and has a low curing temperature (<130 °C). More importantly, it has a high piezoelectric activity (piezoelectric charge coefficient *d*_33_ > 40 pC/N) that exceeds that of other similar materials, such as polyvinylidene fluoride (PVDF), where *d*_33_ is typically between −18 and −30 pC/N. PiezoPaint^TM^ has demonstrated its potential in several applications where thin and flexible ultrasonic transducers are advantageous. For example, PiezoPaint™-based patches have recently been used to deliver low-frequency ultrasound in combination with topical antibiotic application. The device has been shown to be highly effective in killing otherwise resistant biofilm-forming bacteria and thus could be used in the treatment of chronic wounds [18].

Although the use of ultrasound may promote normal healing of the skin, studies have provided conflicting results, and it is still unclear what exact mechanisms underlie the observed effects. Therefore, more fundamental studies at the cellular level are needed to investigate the effects of ultrasound on skin tissue [8]. In general, these effects have been studied using cell cultures and commercially available transducers. However, these transducers are often quite large, are not easily adaptable in terms of operating conditions, and cannot be used simultaneously in multiple culture wells, which can lead to experimental variability [19]. Therefore, ultrasound devices specifically designed for use in cell culture experiments are of great interest.

This paper presents the study of cellular effects of ultrasound generated using a multi-well device based on PiezoPaint^TM^ transducers. The aim is to demonstrate the potential of PiezoPaint^TM^ as a platform to develop therapeutic solutions for the treatment of abnormal scarring. To explore the potential of the technology, human dermal fibroblasts were treated and evaluated in terms of adhesion, proliferation, and deposition of ECM proteins. The secondary aim was to develop a device that could be used to test the effect of ultrasound on cell cultures in vitro, overcoming the limitations of current available transducers.

## 2. Materials and Methods

### 2.1. Fabrication of Ultrasonic Transducers

**Figure 1:**
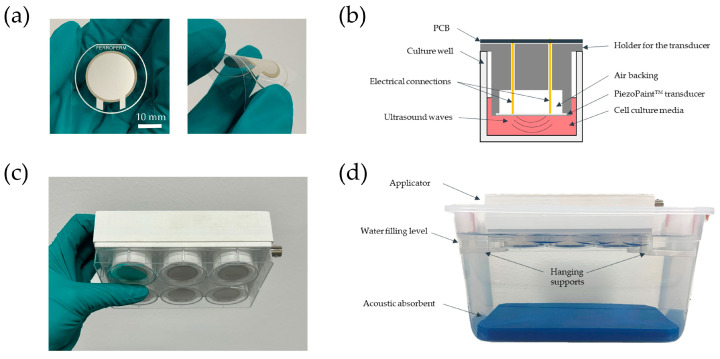
Overview of the multi-well device and experimental setup for ultrasonic treatment. (**a**) Top view of an assembled ultrasound transducer on the substrate of transparent polyethylene terephthalate film (PET) (**left**) and side view of the transducer subjected to bending (**right**); (**b**) Cross-sectional view of a holder for the ultrasound transducer in the device, showing the electrical connections for the transducer to the printed circuit board (PCB), air backing, and placement of the transducers relative to the bottom of the wells. The drawing is not to scale; (**c**) View of a mounted multi-well device on a 6-well plate; (**d**) Side view of the experimental setup showing the placement of a device hanging on top of the water bath and the acoustic absorbent mat at the bottom of the container.

The ultrasonic transducers used in this work were based on a printable piezoelectric material (PiezoPaint™, CTS Ferroperm Piezoceramics, Kvistgaard, Denmark). The transducers consisted of circular sandwich-like structures with a diameter of 23 mm, printed on a thermally stabilized 125 µm thick polyethylene terephthalate film (PET) using a semi-automatic screen printer (M2 K Semi-Auto Screen Printer EKRA, ASYS Group, Dornstadt, Germany). The piezoelectric layer (134.8 ± 1.9 µm thick) was screen printed between a lower and an upper conductive layer (10.3 ± 0.6 µm thick) comprising a flexible polymer-based conductor (AG-530 flexible silver conductor, Applied Ink Solutions, Westborough, MA, USA). An image of an assembled transducer is shown in Figure 1a. The transducers were poled, and the piezoelectric charge coefficient values (*d*_33_) were measured using a piezo-*d*_33_ meter (PM300, PiezoTest, Singapore). Transducers with a *d*_33_ 40.5 + 0.5 pC/N (semi-clamped value) were released for assembly of the multi-well devices.

### 2.2. Design and Fabrication of Multi-Well Devices

The multi-well devices were designed to fit standard six-well culture plates. The devices were fabricated using a fused filament 3D printer (S5, Ultimaker, Utrecht, The Netherlands) with polylactic (PLA) filament (RS PRO 2.85 mm, RS Components, Copenhagen, Denmark). The devices comprised six holders for the ultrasonic transducers. The height of the holders was designed so the transducers were located 1.75 mm from the well bottom when the device was in place (Figure 1b). A printed circuit board (PCB) was fabricated to electrically connect the transducers with spring-loaded electrical pins (pogo-pins). The transducers were wired in parallel to a standard Bayonet Neill–Concelman (BNC) connector mounted onto the PCB, which provided the electrical connection to the driving system. The overall structure was assembled by first gluing each transducer to a holder with ethyl cyanoacrylate (4C10, Permabond, Pottstown, PA, USA). The glue was only in contact with the PET substrate, and the active piezoelectric element was exposed to air backing. A sealing ring was created at the interface between the PET substrate and the external rim of the holder using medical-grade silicone adhesive (Momentive RTV118 FDA, Silicone Solutions Ltd., Bidford on Avon, UK). The PCB was placed on top, and the pogo-pins were inserted through the holes in the holders. A protective case was used to cover the PCB in order to protect it from humidity and to facilitate the manipulation of the devices. Dummy devices were fabricated to use for the controls (no treatment), which comprised multi-well devices of equivalent dimensions but without electrical connections. The assembled multi-well device inserted on a standard 6-well plate is shown in Figure 1c.

### 2.3. Experimental Setup for Ultrasonic Treatment

The multi-well devices were suspended in a plastic container filled with deionized water (Figure 1d). The cell culture plates were partially immersed in water to reduce the ultrasonic reflections that would have occurred at the interface between the plate and the air beneath the plate due to the large difference in acoustic impedance between air (0.00043 MRayl) and the polystyrene comprising the well-plate (2.31 MRayl). The intensity reflection coefficient can be estimated using the equation for plane wave reflections [20]. For an air/polystyrene interface, the reflection coefficient is approximately 99.96%. However, by changing to a water/polystyrene interface, the reflection coefficient decreases to about 21.89%, because water has an acoustic impedance of 1.48 MRayl. An acoustic absorbent (Aptflex F28, Precision Acoustics, Dorchester, UK) was placed at the bottom of the container (Figure 1d). The acoustic absorbent was intended to further minimize formation of standing waves by limiting the reflection of ultrasound waves passing through the bottom of the plate and across the water [21,22]. The piezoelectric transducers were driven with a waveform generator (33521A, Agilent Technologies, Santa Clara, CA, USA) sending a 100 mV, 7 MHz sinusoidal wave, the resonance frequency of the transducers, and a power amplifier with a nominal gain of 50 dB (A075, Electronics & Innovation Ltd., Rochester, NY, USA). The amplitude and frequency set on the waveform generator were chosen to optimize the acoustic response from the transducers and to minimize the temperature rise during exposure.

### 2.4. Experimental Setup for Acoustic Pressure Measurement

The multi-well devices were placed in a glass container filled with deionized water, with the ultrasonic transducer facing up. The acoustic pressure was measured with a hydrophone needle (0.2 mm, Precision Acoustics, Dorchester, UK) placed approximately 2 mm away from the transducers’ surface. The acoustic pressure was mapped over an area of (3 × 3) cm^2^ in 5 mm-steps using a reconfigured 3D printer (Extend 2+ Ultimaker, Utrecht, The Netherlands) to automatically control the position of the hydrophone. The transducers were driven with the same parameters as for the ultrasonic treatment of the cells. 

### 2.5. Temperature Measurements

Since it can be assumed that mechanical and electrical losses in the piezoelectric material will generate heat, an experimental setup was prepared to quantify the temperature increase in the wells caused by the operation of the ultrasonic transducers. Thermocouples (RS PRO Type K, RS Components A/S, Copenhagen, Denmark) were glued to the bottom of a 3D printed replica of a 6-well plate and connected to a data acquisition instrument (34972 LXI, Agilent Technologies). Each well was filled with 2.5 mL of DI water, and the devices were placed in a climatic test chamber (VC3 4034, Votsch Industrietechnik, Balingen-Frommern, Germany) at 37 °C and 95% relative humidity. After the water temperature reached 37 °C, the driving system was switched on, and the temperature increase in each well was recorded after 5, 15, and 30 min.

### 2.6. Cell Culture

Human primary foreskin fibroblasts were obtained from American Type Culture Collection (ATCC nr. CRL 2429, LGC GmbH, Wesel, Germany). Cells were grown in growth medium (GM) consisting of Dulbecco’s modified Eagle Medium (DMEM GlutaMAX, Gibco; ThermoFisher Scientific, Roskilde, Denmark) supplemented with 10% fetal calf serum (FCS), 100 IU/mL penicillin, and 0.1 mg/mL streptomycin (all from ThermoFisher Scientific, Roskilde, Denmark). The cells were maintained in tissue culture flasks (Greiner Bio-one, Frickenhausen, Germany) in a standard humid incubator at 37 °C and 5% CO_2_. The cells were passaged using TrypLE select (Gibco, ThermoFisher Scientific) when they reached 80–90% confluency. All experiments were conducted using cells between passages 3 and 5.

### 2.7. Assessment of Cell Adhesion and Proliferation

To assess the effect of ultrasound treatment on adhesion and growth, cells were seeded in 6-well plates at a density of 5000 cells/cm^2^. To limit cell coverage to the area under the ultrasonic transducers, 500 µL of cell suspension was initially pipetted into the center of each well to allow the cells to settle. After 30 min, the wells were carefully filled with GM and placed in the incubator. After 24 h of incubation, cells were treated with ultrasound for 30 min/day for three days. The cells in the control group (no treatment) were covered with dummy devices. On days 1, 2, and 3 after the start of treatment, cells were fixed and stained for microscopic analysis. The day when the treatment started was considered as day 0. Cell staining followed the protocol described in [23], with minor modifications. Briefly, cells were fixed in 10% formalin, permeabilized with 0.1% Triton X-100, and blocked in 1% bovine serum albumin (BSA). To visualize the cell morphology, F-actin staining was performed by incubation with BODIPY 558/568 phalloidin (B3475, Invitrogen, ThermoFisher Scientific). To study the cell attachment, cells were incubated overnight at 4 °C with focal adhesion kinase antibody (sc-271126, Santa Cruz Biotechnology, Dallas, TX, USA) followed by incubation for 1 h at room temperature with an Alexa Fluor 488-labeled donkey anti-mouse IgG (A-21202, Invitrogen, ThermoFisher Scientific). To quantify the cell growth, nuclei were stained with HOECHST 33342 nuclear stain (Invitrogen, ThermoFisher Scientific). Fluorescence images were obtained using an inverted microscope (AxioObserver.Z1, Carl Zeiss A/S, Birkerød, Denmark) equipped with a digital camera (C11440 ORCA Hamamatsu) and the software package ZEN 2012 (Carl Zeiss A/S). Cell proliferation was estimated by counting the number of nuclei per field in ImageJ (NIH, Bethesda, MD, USA). Quantitative analysis of focal adhesions was performed in ImageJ using the routines described by Horzum et al. [24]. Images of the green channel, containing the focal adhesions, were processed to remove the background and increase the local contrast. Then, a thresholding algorithm was applied to identify the focal adhesions as individual objects in the image. The “analyze particles” command on ImageJ was used to determine the mean area, aspect ratio, and roundness parameter of the focal adhesions in the images. To determine the overall degree of cell coverage (confluency), cells at day 3 were stained using a 0.5% crystal violet solution. The culture plates were imaged using an automatic scanning device (Omni, CytoSMART Technologies, Eindhoven, The Netherlands) with the confluency algorithm to determine the coverage of the cells through each well.

### 2.8. Assessment of Cell Viability

To evaluate the effect of the ultrasound treatment protocol on cell viability, cells were seeded in 6-well plates at a density of 5000 cells/cm^2^. After 24 h, the number of viable cells was determined using a resazurin-based assay (Presto Blue HS, Invitrogen, ThermoFisher Scientific). Following the manufacturer’s instructions, a volume of the Presto Blue reagent, equivalent to 10% of the total medium in the well, was added to the wells. After incubation at 37 °C for 30 min, 100 μL aliquots from each well were transferred in duplicate to a 96-well microtiter plate. Fluorescence was measured at 610 nm with an excitation of 540 nm on a multimode plate reader (EnSpire, PerkinElmer, Waltham, MA, USA). GM was refreshed in all wells, and cells were subjected to ultrasound exposure for 30 min. The dummy device was placed over the cells in the control group. After exposure, the Presto Blue assay was repeated. To estimate the changes in cell viability, a ratio was calculated by dividing the mean fluorescence intensity after exposure by the intensity before exposure. 

### 2.9. Assessment of Extracellular Matrix Deposition

To evaluate the effect of ultrasound on ECM deposition, cells were seeded in 6-well plates at a density of 10,000 cells/cm^2^. After three days, the GM was replaced with an induction medium consisting of GM, supplemented with 0.2 mM L-ascorbic acid (A8960, Sigma-Aldrich, Merck Life Science A/S, Søborg, Denmark). Cells in the treatment group were exposed to ultrasound for 30 min/day, while cells in the control group were covered with dummy devices. After five days, cells in both groups were left in the induction medium for an additional two-day period without exposure. The cell monolayers were treated with a decellularization solution, and the remaining ECM was fixed, and stained using a Sirius Red/Fast Green staining kit (Chondrex Inc., Woodinville, WA, USA) according to the protocol described in [25]. Decellularization was performed with an extraction buffer consisting of 1% Triton X-100 (TX-100) and 20 mM ammonium hydroxide (NH_4_OH) in phosphate-buffered saline (PBS) for 5 min at 37 °C. After a fixation step, staining was performed according to the manufacturer’s instructions. The dye was extracted, and optical densities (OD) were measured at 540 nm and 605 nm using the EnSpire plate reader. Quantitative determination of collagen protein content per well was calculated using the OD values. For morphometric evaluation of the ECM, images of the ECM were acquired before dye extraction using an inverted microscope (AxioObserver.Z1) equipped with a digital camera (AxioCam MRc5) and the software package ZEN 2012 (all from Carl Zeiss A/S). Quantitative analysis of ECM images was performed in ImageJ using the TWOMBLI pipeline described by Wershof et al. with minor modifications [26]. After thresholding the ECM images, the routines of the TWOMBLI package were used to determine two parameters describing the overall ECM patterning: the number of fiber endpoints and the number of fiber branch points per field. In addition, lacunarity scores were calculated as a measure of how the ECM fills the space. 

### 2.10. Assessment of Gene Regulation

Cells for gene regulation analysis were seeded in 6-well plates at a density of 10,000 cells/cm^2^. After three days, the GM was replaced with the induction medium, and cells in the treatment group were exposed to ultrasound for three consecutive days for 30 min/day. On day 1 and 3, cells were lysed, and total RNA was extracted using an Aurum Total RNA isolation kit according to the manufacturer’s instructions (BioRad, Copenhagen, Denmark). cDNA was synthesized using the iScript cDNA synthesis kit (Bio-Rad). Reverse transcription-quantitative polymerase chain reaction (RT-qPCR) was performed in a final volume of 20 µL containing cDNA templates, iTaq Universal SYBR Green Supermix (Bio-Rad), and the appropriate reverse and forward primers for the genes of interest. The reaction was performed on a CFX Connect real-time PCR instrument (Bio-Rad). The reaction was run for 40 amplification cycles, consisting of a DNA denaturation step at 95 °C for 3 min, a temperature hold step for 10 s at 95 °C, and annealing and extension steps at the appropriate annealing temperature for 30 s (Table 1). Genes selected for analysis included *COL1A1*, *COL3A1*, *FN*, and *HSPA1A*. Peptidylprolyl isomerase A (*PPIA*) was used as a control gene to determine the relative expression level in different samples. Primer sequences and annealing temperatures are listed in Table 1.

### 2.11. Statistics

Statistical analysis was performed using Prism v.7 (GraphPad Software, Boston, MA, USA). Two- and multiple-group comparisons were performed using the independent-samples *t*-test and the one-way ANOVA followed by a Tukey post hoc test, respectively. Normality was confirmed using the Shapiro–Wilk test. Homogeneity of variances was assessed using the F-test.

## 3. Results

### 3.1. Acoustic Pressure Measurements and Thermal Effects of Ultrasound Exposure

**Figure 2:**
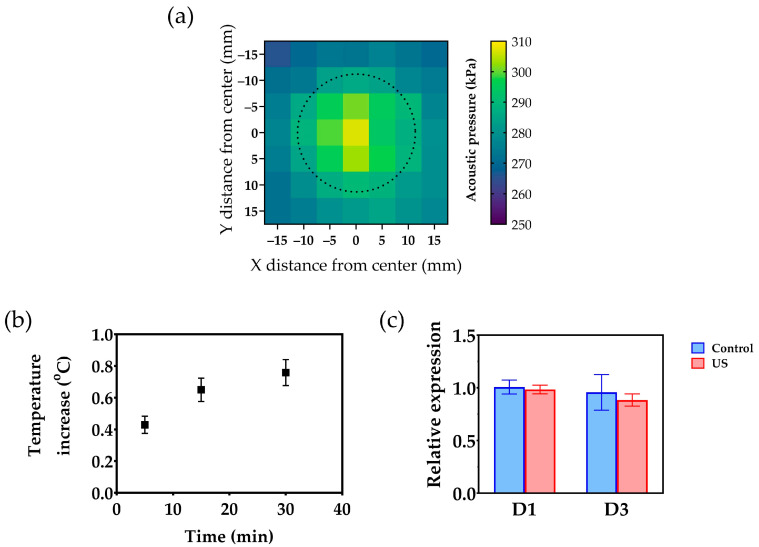
Acoustic pressure measurements and assessment of the thermal effects of ultrasound treatment (US) on the medium and on the cells: (**a**) Heat map depicting the distribution of the average acoustic pressure 2 mm away of a transducer in a multi-well device. The dotted line represents the outline of the ultrasonic transducer. (**b**) Temperature increase in the content of the wells as a function of time. Values are displayed as mean ± SEM (*n* = 24); (**c**) Relative expression of the *HSP1A1* gene, on day 1 (D1) and day 3 (D3). Values are displayed as mean ± SD (*n* = 3).

Acoustic pressure measurements were made using a 49-point array over each transducer of a multi-well device (*n* = 6). The measurements were used to calculate the average acoustic pressure generated by the transducers under operating conditions. Figure 2a shows a heat map depicting the distribution of the average acoustic pressure near a transducer. The pressure was distributed nearly symmetrically from the center and the average peak pressure (306 ± 8 kPa) was at the center of the transducer.

The water contained in the wells showed a steady increase in temperature over time caused by heat generated by mechanical and electrical losses in the piezoelectric material (Figure 1b). After 30 min, the temperature increase reached 0.76 ± 0.08 °C (*n* = 24). To determine whether these temperature changes could induce a significant stress response in cells, the transcriptional activity underlying the expression of Hsp70 was examined in fibroblast cultures. Hsp70 is a 70 kDa heat shock protein, which mediates repair mechanisms that avoid cell damage upon exposure to temperatures well above the physiological range expressed by the *HSPA1A* gene [27]. When the confluent cell layers were intermittently exposed to ultrasound for three consecutive days, the transcriptional activity of the *HSPA1A* gene remained unchanged, both on day 1 and 3 (Figure 1c).

### 3.2. Cell Proliferation and Attachment

**Figure 3:**
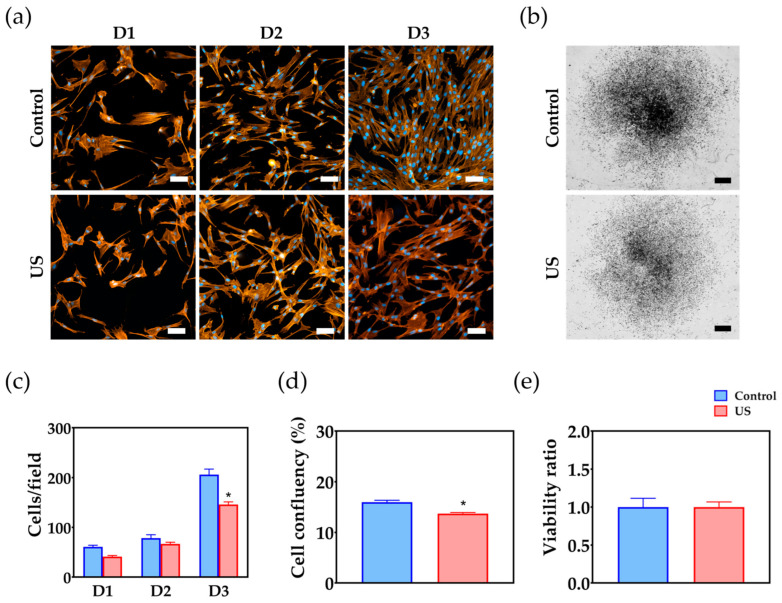
Analysis of cell responses to ultrasound treatment (US): (**a**) Representative images displaying the morphology and distribution of cells in treated and control wells on day 1 (D1), day 2 (D2), and day 3 (D3). Cellular F-actin was stained using BODIPY 558/568 phalloidin (in orange) and nuclei with HOECHST 3342 (in blue). Scale bar = 50 µm; (**b**) Overview images showing the overall distribution of the cells at day 3. Scale bar = 2 mm; (**c**) Quantitative analysis of cell nuclei showing the number of cells over time. Asterisk indicates a statistically significant difference from the control. Values are displayed as mean ± SEM (*n* = 9); (**d**) The graph displays a quantitative estimation of the percentage of coverage of cells in the well. Asterisk indicates a statistically significant difference from the control. Values are displayed as mean ± SEM (*n* = 6); (**e**) Cell viability ratio calculated by dividing the mean fluorescent intensities after exposure by the intensities before exposure. Values are displayed as mean ± SEM (*n* = 6).

On days 1 and 2, cell morphology and density appeared to be unchanged in the treatment group compared to the control group (Figure 3a). However, on day 3, a significant decrease in cell density was observed. These qualitative observations were confirmed quantitatively, as the mean number of cells in the treatment group was significantly lower than in the control group on day 3 (Figure 3c) (*p* < 0.01). Assessment of the global coverage of the wells by cells also confirmed that ultrasound treatment significantly reduced cell proliferation at day 3, as shown in the images (Figure 3b) and quantitative assessment of cell confluency (Figure 3d) (*p* < 0.01). The viability of the cells remained unchanged for both the treated and the control groups (Figure 3e).

**Figure 4:**
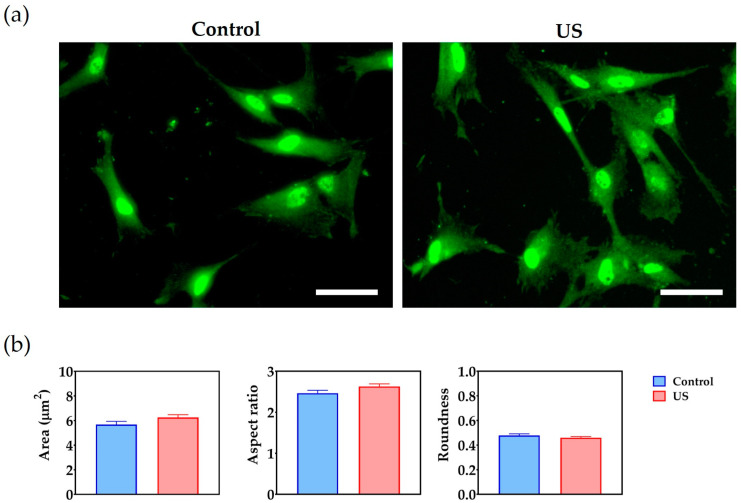
Effect of ultrasound treatment (US) on cell adhesion: (**a**) Focal adhesions are stained with Alexa Fluor 488 (in green). Scale bar = 50 µm; (**b**) Comparative analysis of morphological descriptors of focal adhesions, which include the mean area, aspect ratio and roundness.

Fluorescence microscopy images of the focal adhesions taken on day 1 are shown in Figure 4a. In qualitative terms, ultrasound treatment did not appear to influence the number of focal adhesions or their size. The quantitative analysis supported the qualitative observations, revealing that the average size and geometry of the focal adhesions, estimated using the parameters of aspect ratio and roundness, were not affected by ultrasound treatment (Figure 4b). 

### 3.3. Extracellular Matrix Deposition

**Figure 5:**
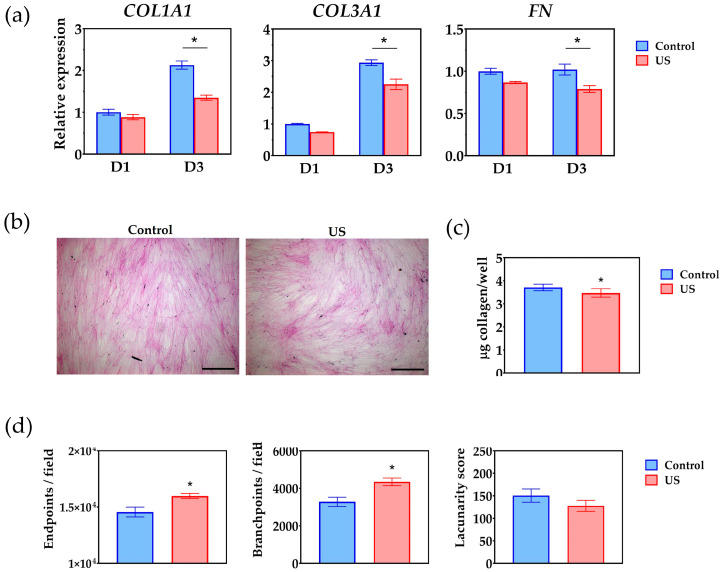
Extracellular matrix (ECM) deposition by cells exposed to ultrasound treatment (US): (**a**) The relative expression of collagen 1 (*COL1A1*), collagen 3 (*COL3A1*), and fibronectin (*FN*) at day 1 (D1) and day 3 (D3); (**b**) Images show ECM stained with Sirius red, with collagenous fibers stained magenta. Scale bar = 200 µm; (**c**) Quantitative measure of the total amount of collagen per well. Values are displayed as mean ± SEM (*n* = 6). Asterisk indicates a statistically significant difference (*p* < 0.01); (**d**) Quantitative description of the ECM morphology by the number of endpoints, branchpoints, and lacunarity score. Values are displayed as mean ± SEM (*n* = 6). Asterisk indicates statistically significant differences (*p* < 0.01).

The effect of ultrasound treatment on ECM deposition was first compared at the level of gene transcription. The relative expression of collagen 1 and collagen 3 followed a time-dependent increase, whereas fibronectin expression levels appeared to be stable between days 1 and 3 (Figure 5a). When assessing the effect of ultrasound on the expression levels, a statistically significant difference was observed at day 3, with the treatment group giving rise to lower expression levels than the control for all genes analyzed (*p* < 0.01). The cells synthesized a complex network of ECM fibers, which was evident after decellularization and staining with the Sirius red/fast green reagent (Figure 5b). While there was no noticeable difference in staining intensity in the microscopic images, quantitative analysis showed a significant decrease in the amount of collagenous protein in the treatment group (*p* < 0.01, *n* = 6) (Figure 5c). Further quantitative analysis using image processing revealed that the parameters representing the complexity of the fiber network (the number of fiber endpoints and branches) were significantly increased in the treatment group (*p* < 0.01, *n* = 6). However, the lacunarity score, which indicates how many gaps or empty spaces are found in the ECM, was not significantly changed by ultrasound treatment (Figure 5d).

## 4. Discussion

The aim of this work was to explore the potential of PiezoPaint™ transducers for the treatment of abnormal scarring by performing an in vitro study in which human dermal fibroblasts were treated and assessed for adhesion, proliferation, and deposition of ECM proteins. The hypothesis was that ultrasound treatment could reduce fibroblast activity in terms of proliferation and ECM deposition without affecting cell viability. For this study, a device was developed that would allow for simultaneous delivery of ultrasound to cells cultured in multi-well plates under standard culturing conditions, while minimizing reflective interfaces near the transducer position that could lead to inconsistencies and non-reproducibility. Abnormal scarring, such as hypertrophic scarring and keloids, are considered as fibroproliferative disorders. Therefore, fibroblasts were used in this study as they in vivo are responsible for initiating collagen production, which is an essential component of the wound healing process which can lead to scarring if disrupted. In normal skin, collagen fibers are composed of both collagens 1 and 3, and an altered ratio between these two collagens could be used to identify abnormalities in the wound healing process. In the context of abnormal scarring, it is therefore relevant to study the effect of ultrasound on fibroblast proliferation and the expression of ECM components, focusing on these two types of collagen [28].

In general, the biophysical effects of ultrasound are divided into thermal and non-thermal effects, which depend, among other things, on the driving parameters of the transducer and modality of the ultrasound waves applied (pulsed or continuous) [29]. Thus, specific parameters can be selected to induce either thermal or non-thermal effects on cells and tissues. Here, the specific exposure parameters were chosen to optimize the acoustic response of the transducers and to minimize the temperature rise during exposure due to thermal effects. It was shown that under the described experimental conditions, only a small temperature increase occurred over time in the wells subjected to ultrasound. In a previous in vitro study aimed at increasing the efficiency of cell transcription by ultrasound, the chosen parameters did not cause a temperature increase above 1.5 °C, suggesting that the main mechanism affecting the transfection efficiency was not thermal [30]. Furthermore, the maximum temperature rise in the current work was far below the temperature values commonly employed to induce heat shock responses in cultured cells. These values are usually above 40 °C and are used, for example, for killing cancer cells with high-frequency focused ultrasound [31,32]. It is well described that preconditioning of dermal fibroblasts with heat or other cellular stressors (such as UV exposure) leads to increased expression of heat shock proteins, such as HSP70 and HSP40 [33,34]. However, the temperature elevations seen here did not appear to induce a significant heat stress response in cells, as reflected by the unaltered transcriptional activity of the heat shock protein HSP70. Therefore, it is possible that the specific parameters used in this study affect cells predominantly via non-thermal mechanisms.

When the effect of ultrasound on the cell behavior was evaluated, a significant effect was found on proliferation, as evidenced by a lower density of fibroblasts at day 3. Studies in the literature have shown varying results regarding the effects of ultrasound on cell growth. While some studies have shown that ultrasound treatment can increase cell proliferation [35], other studies have reported mixed results [36,37] or even no significant effect on proliferation [38]. It is possible that these discrepancies could be due to variability in the experimental design, e.g., in terms of exposure parameters (intensity, frequency, and duration of exposure). Overall, there are several mechanisms that could explain why ultrasound affects cell growth, particularly its effects on cell viability and modulation of the cell cycle [39]. Regarding effects on cell viability, acoustic waves have been shown to produce shear stresses that temporarily open pores in the membrane of mammalian cells, a phenomenon also known as sonoporation [39]. Depending on the dose, ultrasound may cause irreversible damage to the cell membrane, leading to cell death [40]. In the current study, however, cell viability was not significantly affected, so it can be assumed that the chosen exposure parameters did not affect membrane integrity. This is consistent with a study by Duvshani-Eshet and coworkers, in which ultrasound was used to transfect cells by applying a 1.0-MHz ultrasound protocol for up to 30 min, with no significant effect on cell viability detected [41]. On the other hand, the effects on cell cycle progression could be explained by changes in cell adhesion to the substrate, as adherent cells need to spread to progress through the cell cycle [42]. While Zhou and coworkers showed that ultrasound activates integrin receptors associated with focal adhesions, thereby promoting cell proliferation [43], other studies have shown that high intensities may negatively affect cell adhesion, leading to a decrease in the cell growth rate [37]. Morphologic analysis of the focal adhesions in the present work did not show cell morphology or adhesion to the culture surface to be affected by ultrasound exposure. One explanation for this could be that sonication from above has no appreciable effect on cell detachment, which can occur when sonication is applied to the cultures from below [41]. Investigating how different exposure parameters may lead to different cell growth responses is beyond the scope of this study but should be the subject of future research efforts.

Regarding the effects of ultrasound on the ECM deposition, it was found that the synthesis of key ECM components was reduced when cells were exposed to ultrasound. In addition, morphometric analysis of ECM revealed microstructural changes that may be related to disruption of fibril assembly, as ECM matrices in the treatment group were more branched and consisted of shorter segments. Early studies by Harvey and colleagues demonstrated that collagen synthesis in fibroblasts can be differentially modulated by pulsed and continuous ultrasound exposure modalities [44]. A more recent study by Ramirez and colleagues showed that pulsed ultrasound exposure of cultured fibroblasts can cause cell lysis and ultrastructural damage leading to increased collagen synthesis, whereas continuous ultrasound exposure does not appear to affect the rate of synthesis [36]. Ultrasound treatment could also induce local changes in collagen and fibronectin microstructure, especially when applied during the process of fibril self-assembly [45]. The primary focus of this work was not on uncovering the mechanisms that support differential modulation of ECM synthesis and assembly, so further studies are expected in the future to investigate how ultrasound can be exploited to influence interactions between ECM components.

The effect of ultrasound on skin tissue is currently sparsely documented, but is generally described as a double-edged effect that depends on intensity and cell type [46,47]. Therefore, once ultrasound parameters are established, they should be specifically tested not only on fibroblasts but also on other cells associated with skin and wound healing, such as immune cells, keratinocytes, and endothelial cells. Other factors that have been shown to affect normal collagen deposition and lead to abnormal scar formation include fibrin deposition rate, inflammatory cell infiltration, and re-epithelialization and angiogenesis rates [48]. Therefore, the collagen production is central when measuring the mode of action. In addition, complex in vitro or in vivo scar models could be used, as is common in the development of therapies and medical devices. However, the models currently available in this field have numerous drawbacks, most notably the lack of comparability with human skin healing [48]. Therefore, small clinical studies could be of greater value to validate the mode and mechanism of action and estimate the effect size. 

In summary, the data presented here show that ultrasound administered with the multi-well device based on PiezoPaint™ causes a reduction in fibroblast growth and a reduction in ECM deposition compared with non-treated cells. The data suggest that these effects were mediated by non-thermal mechanisms, but they were not mediated by changes in cell viability or adhesion to culture surfaces. Based on the results obtained, it appears that ultrasound treatment may have potential as a means of reducing scarring. Indeed, it is important for an anti-scar treatment to reduce fibroblast proliferation and ECM synthesis [12]. However, it should be noted that these results are based on in vitro studies, and it is uncertain how they translate to in vivo situations where multiple cell types interact in complex ways and blood flow is present. Additionally, the results demonstrate the utility of the proposed device and support its application as a platform for studying cellular responses to therapeutic ultrasound treatment. The main advantages include the ability to expose multiple wells simultaneously and the avoidance of reflective surfaces. It is expected that this device will be a useful tool for mapping the effects of ultrasound treatment on cultured cells, as there is still disagreement about the mechanisms mediating the biological effects of therapeutic ultrasound [49]. Future research should aim to better describe the correlation between exposure modality and effect. In conclusion, the results of this work could contribute to the development of clinically useful wearable devices for the treatment of a variety of skin diseases. The flexibility of the transducers would allow the development of devices that can follow the irregular surface of the human body, which could open a new field of application for scar treatment.

## Figures and Tables

**Table 1:** List of genes, primer sequences, and annealing temperatures (AT) used in this study.

Gene Symbol	Gene Description	Primer Base Sequences (5′-3′)		AT (°C)
		**Forward**	**Reverse**	
*COL1A1*	Collagen type 1 alpha 1	CCT GGA TGC CAT CAA AGT CT	AAT CCA TCG GTC ATG CTC TC	62
*COL3A1*	Collagen type 3 alpha 1	TAC GGC AAT CCT GAA CTT CC	GTG TGT TTC GTG CAA CCA TC	61
*FN*	Fibronectin	ACC TAC GGA TGA CTC GTG CTT TGA	CAA AGC CTA AGC ACT GGC ACA	62
*HSPA1A*	Heat shock protein family A (Hsp70) member 1A	TGT CAG TTC TCA ATT TCC TGT G	GAA ATA GTC GTA AGA TGG CAG T	60
*PPIA*	Peptidylprolyl isomerase A	TCC TGG CAT CTT GTC CAT G	CCA TCC AAC CAC TCA GTC TTG	60

## Data Availability

The data presented in this study are available on request.
